# R-CRISPR: A Deep Learning Network to Predict Off-Target Activities with Mismatch, Insertion and Deletion in CRISPR-Cas9 System

**DOI:** 10.3390/genes12121878

**Published:** 2021-11-25

**Authors:** Rui Niu, Jiajie Peng, Zhipeng Zhang, Xuequn Shang

**Affiliations:** School of Computer Science, Northwestern Polytechnical University, Xi’an 710072, China; nr@mail.nwpu.edu.cn (R.N.); jiajiepeng@nwpu.edu.cn (J.P.); zhipengzhang@mail.nwpu.edu.cn (Z.Z.)

**Keywords:** CRISPR/Cas9, off-target prediction, deep learning

## Abstract

The Clustered Regularly Interspaced Short Palindromic Repeats (CRISPR)—associated protein 9 (Cas9) system is a groundbreaking gene-editing tool, which has been widely adopted in biomedical research. However, the guide RNAs in CRISPR-Cas9 system may induce unwanted off-target activities and further affect the practical application of the technique. Most existing in silico prediction methods that focused on off-target activities possess limited predictive precision and remain to be improved. Hence, it is necessary to propose a new in silico prediction method to address this problem. In this work, a deep learning framework named R-CRISPR is presented, which devises an encoding scheme to encode gRNA-target sequences into binary matrices, a convolutional neural network as feature extractor, and a recurrent neural network to predict off-target activities with mismatch, insertion, or deletion. It is demonstrated that R-CRISPR surpasses six mainstream prediction methods with a significant improvement on mismatch-only datasets verified by GUIDE-seq. Compared with the state-of-art prediction methods, R-CRISPR also achieves competitive performance on datasets with mismatch, insertion, and deletion. Furthermore, experiments show that data concatenate could influence the quality of training data, and investigate the optimal combination of datasets.

## 1. Introduction

The CRISPR-Cas9 (Clustered Regularly Interspaced Short Palindromic Repeats (CRISPR)-CRISPR-associated protein 9 (Cas9)) system is a robust genome-editing tool with a broad range of applications in numerous research [1,2,3]. After the recognition of the 3-nucleotide protospacer adjacent motif (PAM), the endonuclease Cas9 uses a single guide RNA (gRNA) to form base pairs with any DNA target sequences of interest and introduce a site-specific double-strand break [1,4,5]. The high-efficiency and simplicity of CRISPR-Cas9 system enabled genome engineering has great potential in improving agriculture productivity and clinical application [6,7].

The CRISPR-Cas9 system is widely used to enable highly efficient genome editing in various species and cell types, but it may wrongly bind to the unwanted region and cause extra off-target activity. These off-target activities can confound research experiments and also affect the practical application of the technique [8]. The Cas9 can be programmed by altering the sequence of gRNA to target abundant sites in the genome, and the off-target effects of different gRNAs may vary greatly [9]. Therefore, it is crucial to design the off-target prediction model to evaluate the on- and off-target activities of gRNA and choose gRNA with high on-target rate and low off-target effect [10].

From the perspective of gRNA binding to non-target regions, the off-target activities induced by CRISPR-Cas9 mechanism can be divided into three categories: (a) nucleic acid base mismatch with on-target sites; (b) nucleic acid base insertion from gRNA sequence; (c) nucleic acid base deletion from gRNA sequence [11]. The off-target cleavage may occur anywhere in the region that the genome contains a PAM and a protospacer sequence with mismatch, insertion, or deletion. Therefore, accurate evaluation and prediction for the off-target situation of various gRNAs are required for selecting gRNAs with high specificity and targeting accuracy.

The research on off-target prediction models has rose substantial concern in recent years. And the methods that existed mainly include two categories, experimental techniques and in silico methods. Many experimental techniques have been developed such as GUIDE-seq [12], DISCOVER-Seq [13], SMRT-OTS and Nano-OTS [14], Digenome-seq [15,16], CIRCLE-seq [17], CHANGE-seq [18] and target-specific DNA enrichment [19]. Compared with those cell-based techniques that possess the characteristic of high accuracy with high cost, the in silico methods are relatively more convenient and low-cost to predict the off-target activities for particular gRNA without assays.

The early prediction method MIT-score [20,21] figured out that the bases mismatch between gRNA and target DNA follows the sequential-based rules and is highly related to the number and location of bases. Based on the off-target data validated by experiments, MIT-score adjusting the corresponding weights, which allows the discovery of off-target sites in the early stages of gene editing without PAM. Another prediction method based on hand-crafted rules is CCTop [22] considering the distance between off-target sites and PAM since experiments showed that the distance to PAM would affect off-target activities. However, the methods using hand-crafted rules required the manual design of rules, which consumed a lot of effort to adjust the structure and hyperparameters of the network and was dependent on the analysis of the datasets. Furthermore, for those biological structures of sequences that remain unclear, hand-crafted rules may miss extra information.

The first machine learning prediction method CFD [9] proposed by Doench et al. using a lentiviral library infecting MOLM13 cells to obtain a dataset with off-target activities, the experiments showed that CFD outperformed than MIT-score and CCTop. Based on CFD, Listgarten et al. proposed a two-layer regression model Elevation-score [23], which achieved better performance. S.Abadi et al. also presented a regression model CRISTA [24] on the basis of random forest, which referred to the secondary structure of RNA and epigenetic factors in the designing process. Considering the specificity of nucleotide composition and mismatch position on gRNA-target pair, Peng et al. proposed Ensemble SVM [25] to train an ensemble support vector machine classifier. Recently, Wang et al. also presented a generalized prediction method GNL-Scorer [26] to achieve prediction of off-target activities cross-species. For those in silico off-target prediction models based on machine learning, most of them just considered base mismatch and lacked further research on RNA insertion and RNA deletion problems. Meanwhile, those methods cannot mine data features in the best manner and remain limited in prediction accuracy.

The recent application of deep learning to sequence-based problems signifies its applicability on off-target prediction. Chuai et al. implemented DeepCRISPR [27] that combined the epigenetic features and neural network, in which autoencoder and Recurrent Neural Networks (RNN) were utilized to design optimal gRNA as well as predict the on-target and off-target sites simultaneously. Based on Deep Convolutional Neural Network (DCNN) and feedforward neural network, Lin et al. proposed CNN_std [28]. Similarly, Liu et al. also adopted Convolutional Neural Network (CNN) architecture and further introduced attention mechanism into AttnToMismatch_CNN [29]. Another convolutional neural network based on attention mechanism is CRISPR-ONT [30], which paid more attention to a proximal region of PAM that may include cleavage-related information. This method also included a replacement-based sensitivity analysis to illustrate the relative importance of each site. Different from those methods that improved on model architecture, DL-CRISPR [31] focused on dataset optimization. They extended the current positive dataset to improve the competitiveness of the model and investigated dataset design to address data imbalance issue, after that, four layers of CNN were used to learn data features and the final score is got as the average score of 10 models. Recently, Lin et al. proposed CRISPR-Net [32], in which the Inception module that combined several kernels with different sizes were used as feature extractor in the convolutional layer, and the Long Short-Term Memory (LSTM) units were used to form a recurrent neural network in terms of its advantages of selective memory function. Although the method uses a data feature extractor to prevent information loss, it still needs to be further improved to preserve the original information. Meanwhile, since those existing prediction methods cannot satisfy enough precision for implementing CRISPR/Cas9 gene-editing techniques at the clinical level, it is pressing to propose a new method to address the problem.

In this work, we propose an off-target prediction model based on a recurrent convolutional network named R-CRISPR, predicting off-target activities of gRNA-target sequence with mismatch, insertion, and deletion. We first encode the target sequence pair into a binary matrix as the input of the prediction model and then use the preprocessing module on the basis of the RepVGG to extract data features. Finally, the bi-directional recurrent network constructed by Long Short Term Memory units is used for further training of data to improve learning efficiency.

This work provides the following contributions:

1. We developed R-CRISPR, a recurrent convolutional network to evaluate and predict off-target effects of gRNA-target sequence with mismatch, insertion, and deletion.

2. We compare the R-CRISPR with five mainstream prediction methods on datasets obtain from experimental methods to evaluate the model performance. Using the area under the curve of Receiver Operating Characteristic Curve (ROC) and Precision Recall Curve (PRC) as the measurement standard, the performance of R-CRISPR surpasses existing mainstream prediction models.

3. We compare the R-CRISPR with the state-of-art prediction model CRISPR-Net, the R-CRISPR model has an improvement of 0.2% and 1.9% on AUROC and AUPRC.

4. We make extended research to explore the performance difference on various combinations of training datasets, and improve the prediction accuracy by designing an ideal dataset combination.

## 2. Materials and Methods

### 2.1. Datasets

Seven off-target datasets that were validated by mainstream experimental methods were selected for model training and validation [31]. Those datasets were shifted into two categories: one category contains mismatch, insertion, and deletion off-target sites, while another just includes datasets with mismatch off-target sites.

As shown in Table 1, the total sites denote the total number of active off-target sites and inactive off-target sites obtained from Cas-Offinder [33], which search for potential binding targets for Cas9 RNA-guided endonucleases by given gRNA sequences. Dataset CIRCLE contains mismatch, insertion, and deletion, and was confirmed by in vitro method CIRCLE-seq [17]. The highly sensitive unbiased method CIRCLE-seq is based on the principle of detecting new DNA cleavage events, acquired from purified circularized genomic DNA treated with Cas9:gRNA complex, by high-throughput analysis. A total of 7371 off-target sites were validated by CIRCLE-seq with 430 insertion and deletion sites, besides, from the 10 gRNA sequences, Cas-Offinder obtained 577,578 inactive off-target sites.

The datasets in the second category include mismatch sites only. Based on the protein knockout detection method, Dataset PKD was constructed by targeting on human coding sequence CD33 [9], which is composed of 4853 target sites and 2273 off-target sites. Confirmed by Polymerase Chain Reaction (PCR) amplification technology, Digenome-Seq and HTGTS [34], Haeussler et al. constructed Dataset PDH includes 10,129 target sites and 52 off-target sites. Dataset SITE contains 3767 positive off-target sites validated by SITE-seq [35] and 217,733 gRNA-target pairs in total. Tsai et al. constructed Datasets GUIDE_I, GUIDE_II and GUIDE_III [12], Kleinstiver et al. [8] and Listgarten et al. [23] based on the cellular method GUIDE-seq, which required individual transfections for each target. With 294,534 recognized sites in total, Dataset GUIDE_I contains 354 off-target sites with mismatch. And on the basis of the 5 and 22 gRNAs verified by GUIDE-seq, Datasets GUIDE_II and GUIDE_III include 54 and 56 active off-target sites, respectively. In the experimental section, Datasets CIRCLE, PKD, PDH, SITE, GUIDE_I were used as training datasets, while Datasets GUIDE_II and GUIDE_III served as test datasets.

### 2.2. R-CRISPR Model

The construction of R-CRISPR mainly includes three stages. In the first step, the input on-target and off-target sequences are encoded into binary matrices by an encoding scheme. The output of the encoding scheme is then transmitted into a convolutional layer composed of convolutional kernels and RepVGG blocks for data features extraction. Finally, the output of the feature extraction layer is passed to the bi-directional recurrent layers based on LSTM units to learn sequential patterns.

#### 2.2.1. Encoding Matrix Scheme for gRNA-Target Pair

**Figure 1:**
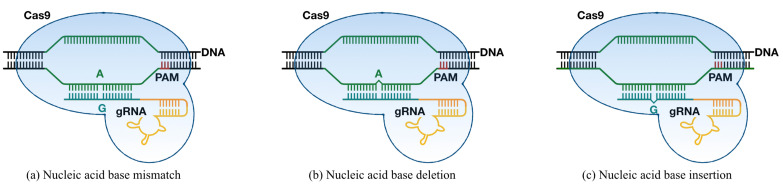
Three categories of off-target activities. (**a**) The situation of base mismatch between guide RNA and target sequence. (**b**) The situation of base deficiency on guide RNA. (**c**) The situation of base insertion on guide RNA.

Suppose $X_{on}=\left[x_{on1},x_{on2},\ldots ,x_{onn}\right]$ could represents the on-target sequences, while $X_{off}=\left[x_{off1},x_{off2},\ldots ,x_{offn}\right]$ demonstrates the off-target sequence, where n denotes the length of sequences. Since the off-target activities could be divided into nucleic base mismatch, nucleic base insertion and nucleic base deletion as shown in Figure 1, $x_{on}$ and $x_{off}$ could be represented by $x_{on},x_{off}\in$ $\left\{A,C,G,T,\_\right\}$ where the symbol “_” denotes insertion or deletion. In terms of the above thoughts, each base in the sequence can be represented by a five-bit vector by one-hot encoding mechanism and the gRNA-target base pair could be encoded into a ten-bit vector as the suitable input of convolutional neural network (e.g., “0100000100” represents the mismatch “C→G”). However, since the off-target sites were analogous to the on-target sites with only difference on mismatch, insertion and deletion site, the encoding scheme could be further optimized.

In the scheme, five-bit vectors $\left(A,C,G,T,\_\right)$ retained the nucleotides of each base pair, and two-bit vectors were used to represent the base pair type (i.e., match, mismatch, insertion, deletion). The seven-bit scheme not only reduces the input size of the neural network but preserves the various information of gRNA-target pairs. Mismatch “T→A” was encoded into “1001001”, where “10010” represented the base pair included A and T, while “01” referred to A as the off-target site and T was the on-target site. Similarly, mismatch “A→T” could be encoded into “1001010”, insertion “_→A” could be presented by “1000101”, and deletion “A→_” could be regarded as “1000110”. As a result, every gRNA-target sequence could be represented by a 7 × 24 matrix $E=\left[x_{1},x_{2},\ldots ,x_{T}\right]$ where 24 is the length of sequence that includes 3-bp PAM adjacent and 21 bases.

#### 2.2.2. Preprocessing Module for Feature Extraction

The classic convolutional neural network VGG [36] achieved excellent performance in image recognition, which uses several 3 × 3 kernels to replace the larger ones and with a simple architecture composed of the convolutional kernel, ReLU activation, and pooling. To improve recognition accuracy, more complicated and well-designed architectures such as ResNet [37], Inception [38] were introduced into the area of computer vision. Though many complicated architectures deliver higher accuracy, there still exist significant drawbacks such as limited implementation and reduce memory utilization.

**Figure 2:**
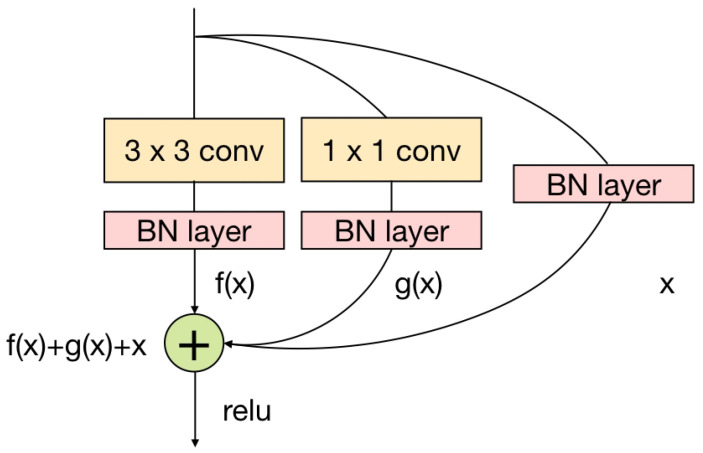
The RepVGG module [39]. A RepVGG module was comprised by a 3 × 3 kernel branch, a 1 × 1 kernel branch and an identity branch.

As shown in Figure 2, a RepVGG [39] block was used as the preprocessing module of R-CRISPR, which had the advantages of multi-branch designs and plain topology designs, to discover useful features and avoid biases introduced by hand-crafted rules. Inspired by ResNet, the structure of RepVGG block includes a 3 × 3 kernel, a 1 × 1 kernel and an identity branch, it becomes y=f(x)+g(x)+x, where f(x) refers to a convolutional kernel with size of 3 × 3, g(x) is a convolutional shortcut implemented by a 1 × 1 kernel, and is an identity branch.

#### 2.2.3. Long Short-Term Memory for Constructing RNN

LSTM [40] is a variant of RNN proposed to solve long-term dependencies problem (i.e., gradient explosion and gradient vanishing) while memorizing long-range information from sequence [41,42]. Meanwhile, LSTM layer is capable of automatically regulating self-connecting loops to memorize long-range information more effectively, since gene sequences could be regarded as the language of biology, such characteristic process significant advantage in learning sequences features.

LSTM composed of two states (i.e., $c_{t}$ and $h_{t}$), and three gates (i.e., input gate $i_{t}$, forget gate $f_{t}$, and output gate $o_{t}$). For each stage, the neuron of neural network provides input $x_{t}$ at time *t*, previous cell state $c_{t-1}$ at time t−1, and previous hidden state $h_{t-1}$ at time t−1. The key equations of LSTM unit are as follows:

$$f_{t}=\sigma \left(W_{f}x_{t}+U_{f}h_{t-1}+b_{f}\right)$$

$$i_{t}=\sigma \left(W_{i}x_{t}+U_{i}h_{t-1}+b_{i}\right)$$

$${\tilde{c}}_{t}=tanh\left(W_{c}x_{t}+U_{c}h_{t-1}+b_{c}\right)$$

$$c_{t}=f_{t}*c_{t-1}+i_{t}*{\tilde{c}}_{t}$$

$$o_{t}=\sigma \left(W_{o}x_{t}+U_{o}h_{t-1}+b_{o}\right)$$

$$h_{t}=o_{t}*tanh\left(c_{t}\right)$$

$$y_{t}=h_{t}$$

where the input sequence is $\left(x_{1},x_{2},\ldots ,x_{T}\right)$, $x_{t}\in R^{d}$, $W\in R^{n\times d}$ and $U\in R^{n\times n}$ refer to the weight matrix, while $h_{t}\in R^{n}$ is the hidden state that uses *n* to represent number of the hidden states, $y_{t}$ is the output at time *t*. Initial value of $c_{0}$ and $h_{0}$ is 0 while the operator “∗” denotes Hadamard product.

#### 2.2.4. R-CRISPR Model Construction

The Long-term Recurrent Convolutional Neural Network (LRCN) that combines CNN and recurrent neural network achieved huge success in the areas of speech recognition and machine translation. Recently, LRCN architecture was also introduced into bioinformatics, and it is approved that the LRCN framework outperformed the CNN and RNN architectures on prediction of transcription factor binding site [33].

**Figure 3:**
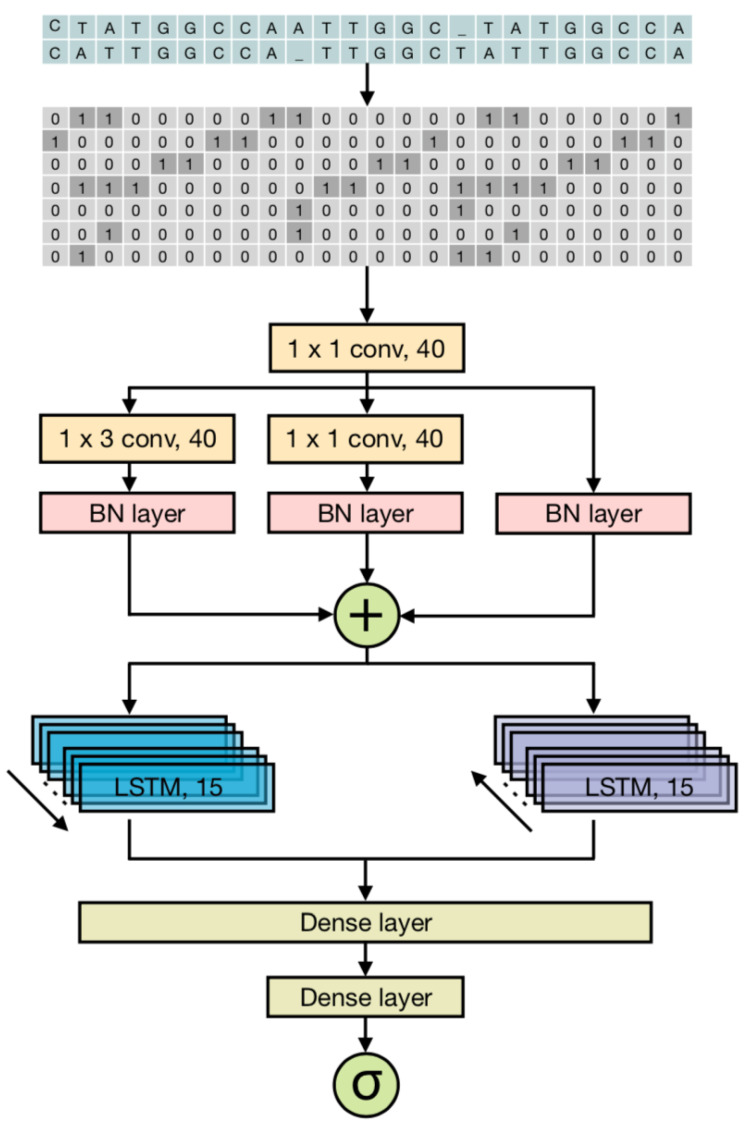
Overview of R-CRISPR. The input on- and off-target sequence pairs is represented into a 7 × length (i.e., length represents the length of gRNA-target sequences) binary matrix as the input of the feature extraction layer. The feature extraction layer contains 40 convolutional kernels and 40 modified RepVGG modules composed of a 1 × 3 convolutional kernel, a 1 × 1 convolutional kernel, and an identity branch. The output of the feature extraction layer is then passed to the bi-directional recurrent layers, each direction is based on 15 LSTM units, to learn sequential patterns of the feature matrix. Followed by the recurrent layer, there are two dense layers with sigmoid as the activation function for final outputs.

Off-target prediction model R-CRISPR was inspired by the LRCN framework and includes an encoding scheme [31] to convert the on- and off-target pair into suitable input for neural network, a convolutional layer, and a recurrent layer. The convolutional layer built on the architecture of CNN and RepVGG [39] module is used as a feature extractor, while the recurrent layer is composed of bi-directional LSTM RNN, and the output of the recurrent layer is passed to the subsequent dense layers. Figure 3 describes the network architecture of R-CRISPR.

On- and off-target sequence pair $\left(X_{on},X_{off}\right)$ was regard as the input of R-CRISPR and passed to the encoding mechanism to be encoded into a binary matrix $E=\left[x_{1},x_{2},\ldots ,x_{T}\right]$ where $x_{t}\in {\left\{0,1\right\}}^{7}$, and *T* referred to the length of the on- and off-target sequence pair. The matrix *E* was then transferred to the convolutional layer comprised forty convolutional filters with size of 1 × 1, learning a representation Δ(·) after convolution and batch normalization operation, and produce matrix $C=\Delta \left(E\right)=\left[c_{1},c_{2},\ldots ,c_{T}\right]$ where $c_{t}\in R^{40}$. was then proceed to the forty RepVGG module that comprised by 3 × 3 kernel, 1 × 1 kernel and identity branch, learning a special representation Φ(·) on *C* and output $R_{i}=\Phi_{i}\left(C\right)=\left[r_{1},r_{2},\ldots ,r_{T}\right]$ where $r_{t}\in R^{40}$. In view of the theory of structural re-parameterization, the input of bi-directional recurrent network $G=R_{1}+R_{2}+R_{3}$ and produce $G=\left[g_{1},g_{2},\ldots ,g_{T}\right]$ where $g_{t}\in R^{40}$.

In order to obtain better analysis of the sequence features extracted by the preprocessing module, the recurrent layer was designed to combine two directional RNNs in which contains 15 LSTM units to learn forward patterns or backward patterns. For the forward direction, each LSTM unit maps the input $g_{t}$ and the previous hidden state $h_{t-1}$ to produce the output $y_{t}^{f}$ and update hidden state $h_{t}$. For the backward direction, each LSTM unit maps the cell state $c_{t}$ and the next hidden state $h_{t+1}$ to produce $y_{t}^{b}$ and update the hidden state $h_{t}$. The output of the current layer combines the outputs of both directions, which refers to $O=\left[\gamma_{1},\gamma_{2},\ldots ,\gamma_{T}\right]$ where $\gamma_{t}={\left[y_{t}^{f},y_{t}^{b}\right]}^{T}$ and $y_{t}^{f},y_{t}^{b}\in R^{n}$. Sigmoid is used as the activation function of the final output neuron after *O* was transferred into the two dense layer with the size of 80 and 20.

The main task of R-CRISPR is to predict the on- and off-target effects, which could be seen as a binary classification task. The labels of the off-target sequence were labeled with label 1, while 0 could represent the other non-off-target sequences. And the Cross Entropy Loss Function was used as the loss function of this model.

$$loss=-\frac{1}{n}\sum \left[y\ln a+(1-y)\ln1-a\right]$$

the *y* refers to the distribution of true label, while the *a* refers to the distribution predicted after training. The Cross Entropy Loss Function could be used to measure the similarity between *y* and *a*, as well as weight update tardiness caused by quadratic loss function when sigmoid is used as activation function.

### 2.3. Mainstream Prediction Methods

In the next experimental section, six mainstream in silico prediction methods will be selected to make a comparison with our model R-CRISPR. As the groundbreaking prediction method based on machine learning, CFD [9] constructed a Naïve Bayes to predict off-target activities and surpassed the hand-crafted rules models. The widely recognized regression model Elevation-score [23] contained two layers, the first layer using boosted regression tree to predict the off-target score for single mismatch of gRNAs, while the second layer constructed an L1-regularized linear regression combiner model to calculate the aggregate score of a single gRNA from multiple off-target activities related to it. With the same accuracy, the training speed of Ensemble SVM [25] was greatly improved, which made it comparatively more suitable for large datasets. In CNN_std [28], each sequence was encoded into a matrix as input firstly, and then multiple sizes of filters were used to capture features in different ranges, the feature matrix was passed several convolutional layers and a dense layer to learn sequential patterns. Also based on CNN architecture, AttnToMismatch_CNN [29] introduced an attention mechanism to select the information that was highly correlated to off-target activities as whole gRNA-target sequence information. The state-of-art prediction model CRISPR-Net [32] combined the advantages of the inception module and LSTM units, which had achieved higher performance accuracy than previous models.

## 3. Results

In the training time, Adam optimizer dynamically optimized the learning rate to achieve both efficiency and effectiveness and the initial learning rate of weight was set as 0.0001. Besides, the batch size of each batch was set at 10,000 with the epoch number was set as 100. To systematically represent the performance of prediction models, ROC (Receiver Operating Characteristic curve) and PR (Precision-Recall) analysis were used as an evaluation criterion. As Table 2 shows, the hyperparameters are as follows:

Besides, all components of R-CRISPR were implemented using Keras 2.2.4 with TensorFlow 2.3.0 backend.

### 3.1. Performance of R-CRISPR on Mismatch-Only gRNA-Target Prediction

In terms that base mismatch occupies a large proportion in three kinds of off-target types (i.e., nucleic acid base mismatch, nucleic acid base insertion, and nucleic acid base deletion), and the existed mainstream prediction methods were designed to predict off-target sites with mismatch, we first verified the performance of R-CRISPR with six models (i.e., AttnToMismatch_CNN, Elevation-score, CFD, Ensemble SVM, CNN_std and CRISPR-Net) on mismatch-only datasets. Using the combination of datasets PKD, PDH, and GUIDE_I as training set and tested on dataset GUIDE_II. R-CRISPR achieved relatively highest performance both AUROC (Area under ROC curve) and AUPRC (Area under PR curve), with an accuracy of 0.991 on AUROC and 0.319 on AUPRC. As shown in Table 3, the difference is relatively slight on AUROC while AUPRC score appeared significant differences between diverse models, the R-CRISPR appeared maximum value of 0.319, and minimum value is 0.071 of AttnToMismatch_CNN. Though the AUROC score of R-CRISPR (0.991) was slightly lower than Ensemble SVM (0.993) and CRISPR-Net (0.993) on the GUIDE-seq dataset, R-CRISPR held an improvement of 18.8% and 2.7% on AUPRC.

### 3.2. Performance of R-CRISPR on Multiple gRNA-Target Prediction

In the previous section, we had evaluated the performance of R-CRISPR on mismatch-only datasets and proved that R-CRISPR outperformed the six existing models in the previous experiment, during the second stage, we explored how nucleic acid base insertion and deletion affect prediction accuracy and made comparison with the state-of-art off-target prediction method CRISPR-Net. CRISPR-Net is built upon a long-term recurrent convolutional neural network and could recognize off-target activities with base mismatch, insertion and deletion. Moreover, Elevation-score was served as a benchmark to better evaluate model performance.

**Figure 4:**
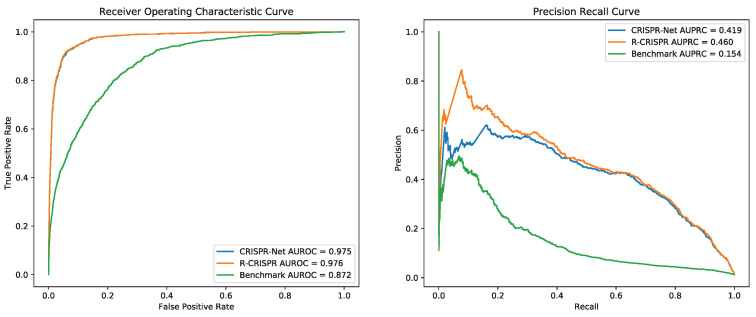
Comparison of CRISPR-Net and R-CRISPR on 5-fold Cross Validation with Dataset CIRCLE.

Since Dataset CIRCLE was the only dataset that contained three categories of off-target activities, three models were evaluated with 5-fold cross-validation. For each validation, one subset was used as the test dataset and the other four subsets were served as the training dataset. Figure 4 shows that compared with CRISPR-Net, though CRISPR-Net represented a tiny higher accuracy on AUROC (0.1%), R-CRISPR achieved an improvement of 4.1% on AUPRC.

**Figure 5:**
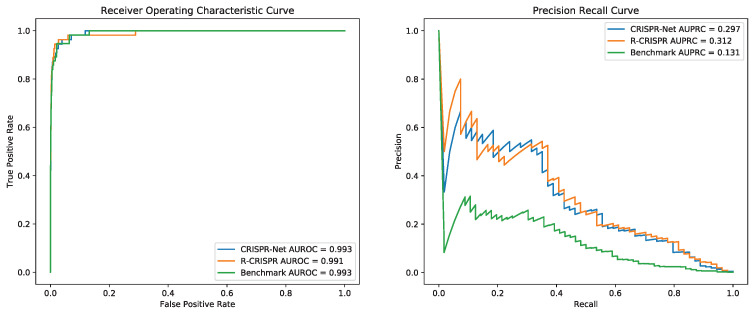
Comparison of CRISPR-Net and R-CRISPR on Dataset GUIDE_II.

Combination of datasets CIRCLE, PKD, PDH, SITE, and GUIDE_I as training datasets by concatenating, to preserve the biological information of insertion and deletion while adding more mismatch sites. As shown in the Figure 5, drafting ROC curve and PR curve based on the prediction result on dataset GUIDE_II, R-CRISPR (AUROC = 0.991, AUPRC = 0.312) outperformed than CRISPR-Net (AUROC = 0.993, AUPRC = 0.297) on AUPRC with an improvement of 2.2%, and also surpassed the Benchmark (AUROC = 0.993, AUPRC = 0.131) on AUPRC with an improvement of 18.1%. Furthermore, we believe that the data concatenate may affect the quality of training data and improve model performance. Thus, we investigated various combinations of datasets to improve the performance of R-CRISPR in the next section.

### 3.3. Performance of R-CRISPR with Different Training Datasets

In previous study, we figured out that the model predictive performance could be influenced by the quality of training datasets. Thus, we generated seven training datasets (see Table 4) from five experimental datasets (i.e., datasets CIRCLE, PKD, PDH, SITE and GUIDE_I) in which the active off-target sites were validated by CIRCLE-seq, Digenome-seq, SITE-seq and GUIDE-seq.

**Figure 6:**
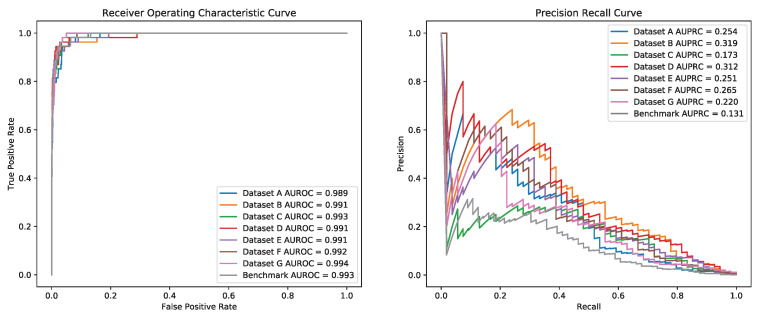
Performance of R-CRISPR with different training datasets on Dataset GUIDE_II.

Testing on dataset GUIDE_II, seven R-CRISPR models represented competitive performance on the ROC curve as shown in Figure 6, with an average AUROC of 0.992. However, the test results of seven training datasets were numerous on the PR curve, dataset B (Combination of datasets PKD, PDH, SITE and GUIDE_I) achieved the highest AUPRC of 0.319, while dataset C (Only includes dataset CIRCLE appeared the lowest AUPRC score of 0.173. The result indicated that the designing of training dataset could improve predictive performance significantly, which may be because those datasets achieved higher accuracy also contains more abundant gRNAs and off-target sites. The R-CRISPR trained on combined datasets surpassed those trained on a single dataset among all seven models on dataset GUIDE_II. The model trained on dataset B reached the highest AUPRC of 0.319, which possessed an advantage of 0.7% on AUPRC compared to the second best model (AUROC = 0.991, AUPRC = 0.312) trained on dataset D (Combination of dataset CIRCLE, PKD, PDH, SITE and GUIDE_I), and had an improvement of 5.4% on AUPRC compared to the third best model (AUROC = 0.992, AUPRC = 0.265) trained on dataset F (Combination of dataset PKD, PDH, and GUIDE_I).

**Figure 7:**
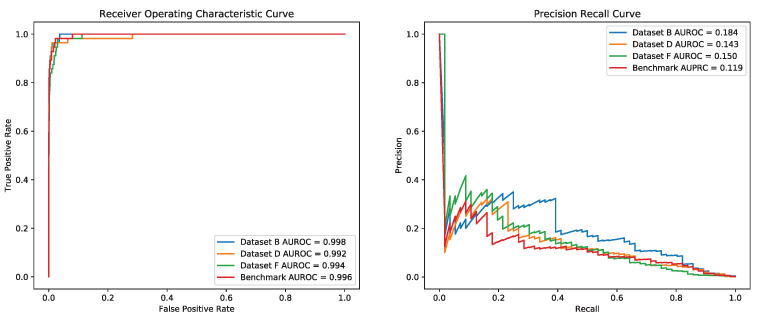
Performance of R-CRISPR with different training datasets on Dataset GUIDE_III.

As shown in Figure 7, For further exploring the efficiency of models trained on datasets B, D and dataset F, we tested those models on dataset GUIDE_III in which concludes 56 off-target sites and 22 diverse gRNAs. Table 5 shows it is obvious that the model trained on dataset B could achieve better performance (AUROC = 0.998, AUPRC = 0.184), and appeared an improvement of 0.4% and 3.4% on both AUROC and AUPRC than the second best model (AUROC = 0.994, AUPRC = 0.150) trained on dataset F.

### 3.4. Hyperparameters Optimization

The optimization process of large-scale machine learning usually contained a large number of hyper parameters that needed to be fixed by users according to a certain application, and the design of hyper parameters could directly influence the model performance. In this optimization section, we would like to explore hyperparameters combination that could achieve higher performance based on five kinds of hyper parameters (i.e., dropout_rate, learning_rate, batch_size, and epochs).

**Figure 8:**
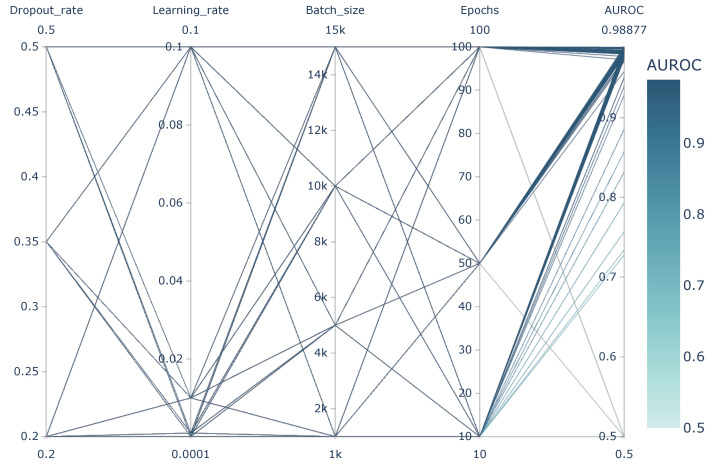
Performance of R-CRISPR with different combinations of hyperparameters.

Given a set of hyperparameters and the potential assignments from its parameter space, the fundamental method Grid Search was used as the search practice to select the combination that outperformed others. Furthermore, we selected the Dataset CIRCLE as test data since it contains most off-target activities as well as various off-target categories, and AUROC was used to evaluate the certain performance of hyperparameters combinations. As Figure 8 shows, the best combination achieved 0.98877 on AUROC, in which dropout_rate = 0.5, learning_rate = 0.001, batch_size = 10,000 and epochs = 50. Significantly, learning_rate was inappropriate to set too high, while dropout_rate and epochs were not suitable to be too low.

## 4. Discussion

The accurate evaluation of off-target activities in the CRISPR-Cas9 system is a severe issue when applying machine learning. Since the early prediction models remained hand-crafted rules and limited predictive accuracy. In this study, we first used an encoding scheme to encode each gRNA-target sequence into a 7 × 24 matrix as the input of an improved convolutional neural network for data feature extraction. Then, given the above strategies, we proposed R-CRISPR, an off-target prediction model based on a recurrent convolutional network with a Cross Entropy Loss Function to solve the problem. Since the mainstream in silicon off-target activities prediction methods lacked further research on gRNA-target pairs insertion and deletion problems, we optimized R-CRISPR to satisfy the demands of insertion and deletion detection. We first explored the prediction accuracy of mismatch problems in terms that nucleic acid base mismatch occupies the main proportion of off-target sites and most existing predictive methods were designed for mismatch-only problems. On mismatch-only off-target dataset GUIDE_II verified by GUIDE-seq, experiments show that R-CRISPR outperformed six existing mainstream predictive methods on both ROC and RC analysis with an average accuracy of 0.991 on AUROC and 0.319 on AUPRC. In addition, we set a 5-fold cross-validation test based on the off-target dataset confirmed by CIRCLE-seq (with nucleic acid base insertion and deletion) to investigate how insertion and deletion problems affect the off-target prediction. We trained and compared R-CRISPR with the state-of-art prediction method CRISPR-Net, which could also measure off-target sites with insertion and deletion, on different combinations of datasets. R-CRISPR achieved a higher accuracy of 0.976 on AUROC and 0.460 on AUPRC with an improvement of 0.1% and 4.1% than CRISPR-Net. Furthermore, we also explored how the quality of training data is influenced by data concatenation and designed seven combinations of datasets to test the performance of R-CRISPR. Seven R-CRISPR models expressed competitive performance on ROC analysis with an average accuracy of 0.992 on AUROC, while the test results were numerous on PR analysis with the highest accuracy achieved 0.319 and lowest one appeared 0.173. The experiments indicated that the designing of training datasets could affect predictive results significantly, and the R-CRISPR trained on combined datasets surpassed those trained on a single dataset. We believed that the combination of multiple datasets could obtain multifarious information of off-target activities, and produce a more comprehensive dataset, hence improving the model performance. Meanwhile, we speculated that the sample imbalance caused by fewer positive samples was also a crucial point for model performance. Since the off-target activities only occupied a minority number in the whole biological process, the datasets obtained from most experiments were unbalanced, which required further optimization.

## 5. Conclusions

In our work, we developed R-CRISPR to contribute to the quantification of off-target activities with nucleic acid base mismatch and deletion problems. The architecture of R-CRISPR demonstrated the practicality of convolutional recurrent neural network in predicting off-target sites between gRNA sequence and target DNA sequence. Since convolutional network could be used to do preliminary information extraction, we applied the RepVGG module in the convolutional layer to capture features for the target sequence with unclear biological structure, and combined a bi-directional recurrent network based on LSTM units for further training. Furthermore, as complementary off-target sequences and related datasets become available, the efficiency and predictive accuracy are expected to be improved. We will also carefully investigate more superior model architecture based on deep learning and an optimized combination of training datasets to improve model performance. In a nutshell, the experimental results in our work fully demonstrated that R-CRISPR is an effective off-target prediction method and can contribute to the gRNA design in the CRISPR-Cas9 system.

## Figures and Tables

**Table 1:** Experimental datasets details validated by different methods.

Dataset	Train	Test	Total Sites	Off-Target Sites	Insertion/Deletion	gRNAs
CIRCLE	√	-	584,949	7371	430	10
PKD	√	-	4853	2273	-	65
PDH	√	-	10,129	52	-	19
SITE	√	-	217,733	3767	-	9
GUIDE_I	√	-	294,534	354	-	9
GUIDE_II	-	√	95,829	54	-	5
GUIDE_III	-	√	383,463	56	-	22

**Table 2:** Hyperparameters settings in training time.

Hyperparameter	Value
Weight optimizer	Adam optimizer
Weight learning rate initialization	0.0001
Batch size	10,000
Epoch	100

**Table 3:** Performance of seven off-target prediction methods on Dataset GUIDE_II.

Off-Target Prediction Methods	AUROC	AUPRC
AttnToMismatch_CNN	0.961	0.071
CRISPR-Net	0.993	0.292
Elevation-score	0.993	0.131
CFD	0.925	0.066
Ensemble SVM	0.982	0.113
CNN_std	0.956	0.115
R-CRISPR	0.991	0.319

**Table 4:** Performance of R-CRISPR with different training datasets on Dataset GUIDE_II.

Training Dataset	CIRCLE	PKD	PDH	SITE	GUIDE_I	AUROC	AUPRC
A	√	√	√	-	√	0.989	0.254
B	-	√	√	√	√	0.991	0.319
C	√	-	-	-	-	0.993	0.173
D	√	√	√	√	√	0.991	0.312
E	-	-	-	√	-	0.991	0.251
F	-	√	√	-	√	0.992	0.265
G	√	-	-	√	-	0.994	0.220
Benchmark	√	√	√	√	√	0.993	0.131

**Table 5:** Performance of R-CRISPR with different training datasets on Datasets GUIDE_III.

Training Dataset	CIRCLE	PKD	PDH	SITE	GUIDE_I	AUROC	AUPRC
B	-	√	√	√	√	0.998	0.184
D	√	√	√	√	√	0.992	0.143
F	-	√	√	-	√	0.994	0.150
Benchmark	√	√	√	√	√	0.996	0.119

## Data Availability

Data used in this article was obtained from Jiecong Lin, and they are available at https://codeocean.com/capsule/9553651/tree/v1 (accessed on 24 September 2021).

## Abbreviations

The following abbreviations are used in this manuscript:

CNN	Convolutional Neural Network
Cas9	CRISPR-associated protein 9
CRISPR	Clustered Regularly Interspaced Short Palindromic Repeats
DCNN	Deep Convolutional Neural Network
gRNA	guide RNA
LRCN	Long-term Recurrent Convolutional Neural Network
LSTM	Long Short-Term Memory
PAM	protospacer adjacent motif
PRC	Precision Recall Curve
RNN	Recurrent Neural Networks
ROC	Receiver Operating Characteristic
